# Outcomes with multi-disciplinary management of central lung tumors with CT-guided percutaneous high dose rate brachyablation

**DOI:** 10.1186/s13014-021-01826-1

**Published:** 2021-06-07

**Authors:** Stephanie M. Yoon, Robert Suh, Fereidoun Abtin, Drew Moghanaki, Scott Genshaft, Mitchell Kamrava, Alexandra Drakaki, Sandy Liu, Puja Venkat, Alan Lee, Albert J. Chang

**Affiliations:** 1 Department of Radiation Oncology, University of California Los Angeles, 200 Medical Plaza Driveway, Suite B265, Los Angeles, CA 90095 USA; 2 Department of Radiology Thoracic Interventional Services, University of California Los Angeles, Los Angeles, CA 90095 USA; 3 Department of Radiation Oncology, Veterans Affairs Greater Los Angeles, Los Angeles, CA 90073 USA; 4 Department of Radiation Oncology, Cedars-Sinai Medical Center, Los Angeles, CA 90048 USA; 5 Department of Hematology and Medical Oncology, University of California Los Angeles, Los Angeles, CA 90095 USA

**Keywords:** Brachytherapy, Interstitial brachytherapy, High-dose-rate brachytherapy, Brachyablation, Pulmonary metastasis

## Abstract

**Background:**

Centrally located lung tumors present treatment challenges given their proximity to mediastinal structures including the central airway, esophagus, major vessels, and heart. Therapeutic options can be limited for medically inoperable patients, particularly if they have received previous thoracic radiotherapy. High dose rate (HDR) brachyablation was developed to improve the therapeutic ratio for patients with central lung tumors. The purpose of this study is to report initial safety and efficacy outcomes with this treatment for central lung malignancies.

**Methods:**

From September 2015 to August 2019, a total of 25 patients with 37 pulmonary tumors were treated with percutaneous HDR brachyablation. Treatment was delivered by a multi-disciplinary team of interventional radiologists, pulmonologists, and radiation oncologists. Twenty-three patients received a median dose of 21.5 Gy (range 15–27.5) in a single fraction, whereas two patients received median dose of 24.75 Gy (range 24–25.5) over 2–3 fractions. Tumor local control (LC) was evaluated by Response Evaluation Criteria in Solid Tumors v1.1. Treatment-related toxicities were graded by Common Terminology Criteria for Adverse Events v5.0, with adverse events less than 90 days defined as acute, and those occurring later were defined as late. LC, progression-free survival (PFS), and overall survival (OS) rates were estimated by the Kaplan–Meier method.

**Results:**

Of 37 treated tumors, 88% were metastatic. Tumor location was central and ultra-central in 24.3% and 54.1%, respectively. Average tumor volume was 11.6 cm^3^ (SD 12.4, range 0.57–62.8). Median follow-up was 19 months (range 3–48). Two–year LC, PFS, and OS were 96.2%, 29.7%, and 65.5%, respectively. Thirteen of 39 (33.3%) catheter implantation procedures were associated with trace minor pneumothorax requiring no intervention, 1 (2.5%) procedure with minor radiographic pulmonary hemorrhage, and 4 (10.3%) with major pneumothorax requiring chest tube insertions. All procedural complications resolved within 24 h from treatment. Acute grade 1–2 toxicity was identified in 4 patients, whereas none developed late toxicity beyond 90 days of follow-up.

**Conclusion:**

Percutaneous HDR brachyablation is a safe and promising treatment option for centrally located primary and metastatic lung tumors. Future comparisons with stereotactic body radiotherapy and other ablative techniques are warranted to expand multi-disciplinary management options.

## Introduction

The uncontrolled growth of central tumors can contribute to significant morbidity, including hemoptysis, lung collapse, vascular obstruction, and dysphagia. Yet, treatments can be challenging given their close proximity to critical mediastinal structures including the central airway, esophagus, major vessels, and heart. Patients who are deemed poor candidates for surgery are often recommended external beam radiotherapy. Whenever long-term control is desired, short courses of stereotactic body radiotherapy (SBRT) can achieve local control (LC) rates of 80–90% [1, 2]. However, SBRT to centrally located tumors has been associated with severe toxicities [2–6], which has led to the development of alternative strategies such as image-guided thermal ablation (IGTA) that has been associated with variable levels of LC and complications related to applicator placement and delivery of thermal energy [7–9].

Despite such challenges, local control of centrally located tumors is critical for palliation and to improve quality of life. Moreover, treatment of patients with limited metastases for curative intent is increasing [10–12]. This oligometastatic paradigm involves strategic combinations of locoregional therapies and effective systemic agents.

To develop a potentially safer and more effective treatment strategy for central lung tumors, our institution introduced percutaneous high dose rate (HDR) brachyablation for lung tumors in 2015The term “brachyablation” is commonly referenced at our institution to facilitate communication with our multi-disciplinary colleagues who are familiar with radiofrequency ablation, microwave ablation, and cryoablation. To our knowledge, our institution was the first to offer percutaneous brachyablation in the United States for pulmonary tumors. This initial study reports the safety and efficacy of this treatment in the initial 25 patients treated with this technique.

## Materials and methods

A retrospective cohort study of the first 25 consecutive patients with pulmonary tumors treated with CT-guided interstitial HDR brachytherapy from September 2015 to August 2019 was conducted at a single institution after institutional review board approval. Central tumors were lesions located within 2 cm from the proximal bronchial tree or mediastinum; tumors whose CTV abutted the aforementioned structures were considered ultra-central. Patients had been considered for treatment in a multi-disciplinary setting if they had biopsy proven primary non-small cell lung cancer (NSCLC), locally recurrent NSCLC, or metastatic pulmonary tumors confirmed with growth on imaging, who were medically inoperable, not surgical candidates, or refused surgery.

### Interstitial catheter implantation

**Fig. 1:**
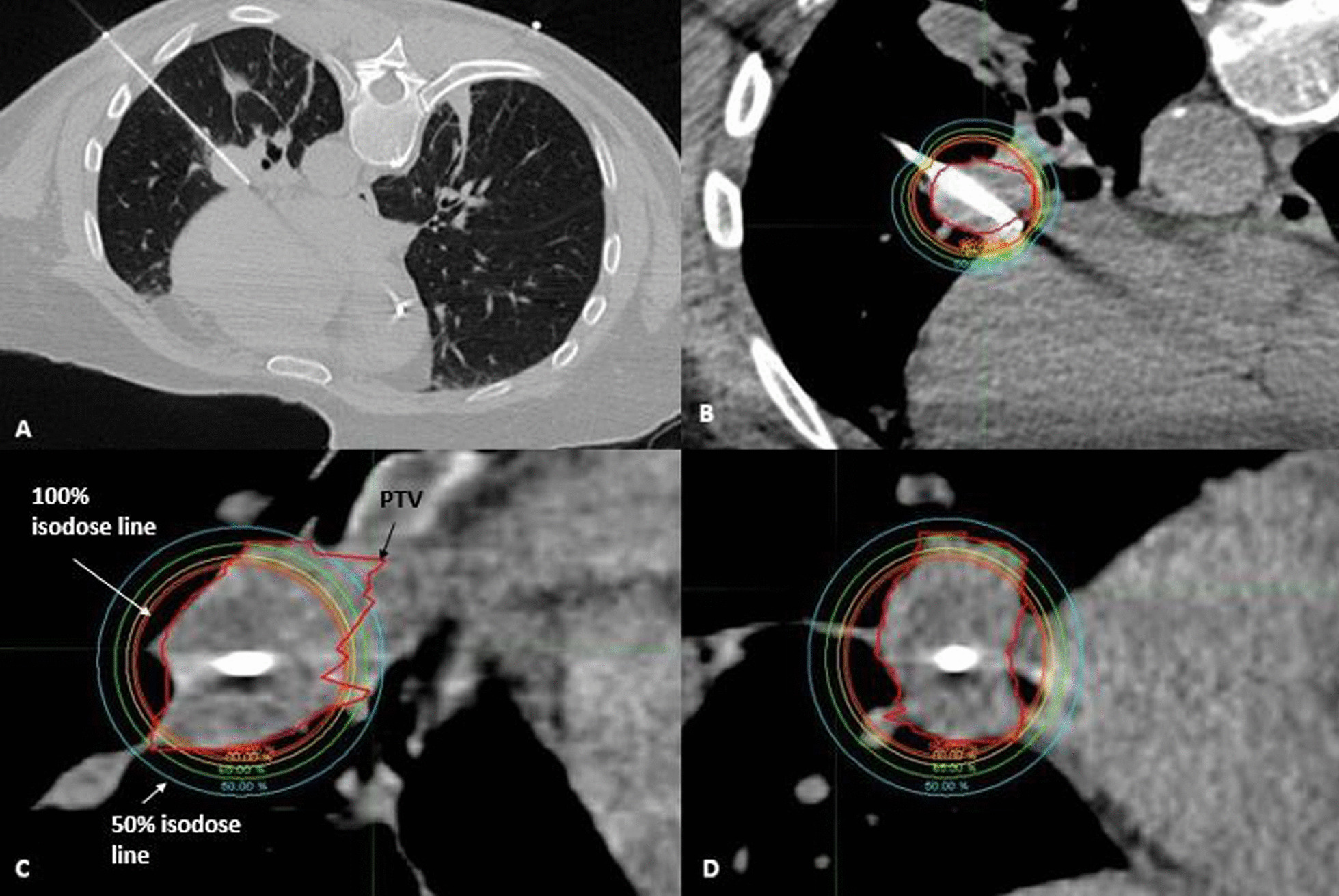
A 60-year old man with metastatic leiomyosarcoma presenting with multiple lung metastasis. He had undergone multiple microwave ablations for other lung tumors. A malignant left sub-hilar lymph node continued to grow despite treatment with multiple cycles of doxorubicin and olaratumab. This tumor was treated with CT-guided interstitial HDR brachyablation. **A** Placement of co-axial needle under CT-guidance during brachytherapy catheter implantation. A co-axial needle was advanced percutaneously and placed directly into the ultra-central tumor abutting the heart. **B** Axial **C** Sagittal and **D** Coronal views of resultant treatment isodose distribution

Catheter placements were performed in collaboration with the Department of Radiology Thoracic Interventional Services. With exception of one patient, all patients were treated on an outpatient basis. At the onset of their procedure, patients underwent a non-contrast CT for tumor localization, and a mark was placed on the overlying skin. Local anesthesia with lidocaine (2%) was administered to the skin surrounding the marked site, and bupivacaine (0.5%) provided deeper soft tissue and pleural anesthesia. Patients received conscious sedation according to individual needs. A single 17-gauge co-axial needle (Argon Medical Devices, Athens, TX, USA) was inserted percutaneously through the marked location using image guidance with serial CT scans to confirm accurate needle trajectory as the needle tip advanced to the distal or deep margin of the targeted tumor (Fig. 1A). A single 4F brachytherapy catheter (Best Medical International Inc., Springfield, VA, USA) was introduced through the co-axial needle until both tips were coincident with each other intratumorally. Additional needles and catheters were introduced as needed to ensure adequate dosimetry of the tumor, especially when tumor diameter exceeded 3 cm and/or when tumor shape was non-spherical.

### Brachytherapy planning and treatment delivery

A planning CT simulation scan using 2 mm slice thickness was obtained. Images were transferred to the brachytherapy treatment planning system (TPS) (Oncentra Brachytherapy, version 4.5.2, Elekta Inc., Veenendaal, Netherlands). Catheters were digitized and reconstructed on the TPS. The clinical target volume (CTV) was delineated on the simulation scan, which included the gross tumor and suspicious areas from prior diagnostic scans. Critical organs-at-risk (OAR) were contoured on each slice. Inverse-planning was utilized, and a prescription range of 15 to 27.5 Gy was delivered to the CTV periphery in a single treatment fraction corresponding to a biologic effective dose (BED_3_) ≥ 100 Gy (Fig. 1B–D). Two patients (8%) were treated with multiple fractions to deliver a median dose of 24.8 Gy (range 24.0–25.5); one patient underwent 2 separate outpatient catheter implantations for each fraction, and another had one catheter implantation and hospitalized overnight to receive 3 treatment fractions in 4–6-h intervals. Dose fractionation schemas were compared on common numeric scores using BED_3_ and equivalent dose in 2 Gy per fraction (EQD2) (Additional file 1: Appendix). A minimum of  95% of the CTV was to receive the prescription dose (V100% > 95%) and the dose that 90% of the CTV received (D90%) was optimized. OAR dose tolerance limits were prioritized as outlined by AAPM Task Group 101 [13]. Maximum dose, corresponding to the most irradiated 2 cc of the heart, esophagus, trachea, proximal bronchial tree, and chest wall (D2cc) as well as ipsilateral lung V5Gy, V20Gy, and mean dose were recorded. Iridium-192 was delivered with an HDR remote afterloader (Nucletron B.V., Model 136149A02 Flexitron HDR remote afterloader, Elekta Inc., Veenendaal, Netherlands). After treatment, both the catheter(s) and co-axial needle were removed with placement of a resorbable hydrogel (BioSentry Tract Sealant System, Surgical Specialties Corp., Tijuana, MX) to seal the pleural site of entry. A final CT scan was acquired after catheter removal.

### Follow-up and statistical analysis

Patients were routinely followed within 7 days of treatment and at least every 3 months with surveillance chest CT scans. Target lesions were assessed by the Response Evaluation Criteria in Solid Tumors (RECIST) by two independent radiation oncologists and a thoracic radiologist [14], and scored as stable disease, partial response, or complete response. Growth of the treated tumor compared to previous CT scans was considered as local failure. Toxicities were graded by Common Terminology Criteria for Adverse Events (CTCAE) version 5.0; toxicities less than or equal to 90 days after treatment were defined as acute, and those occurring afterwards were defined as late.

Descriptive statistics were reported for patient demographics, clinical features, procedural complications, and toxicity. Fisher’s exact test assessed the association between LC and several covariates: implantation setting (non-metastatic vs. metastatic), prior IGTA, lung radiation, systemic therapies, tumor location, and total dose (< 20 Gy vs. ≥ 20 Gy). Time-to-event analysis was performed with the endpoint defined as time from the start of first percutaneous HDR brachyablation procedure. Endpoints were time to local failure, disease progression (local, regional, or distant), and death. Tumor LC, progression free survival (PFS) and overall survival (OS) rates, respectively, were estimated using the Kaplan–Meier method. All analyses were conducted using Stata IC version 15 (StataCorp, College Station, Texas, USA). This study followed the Strengthening the Reporting of Observational Studies in Epidemiology (STROBE) reporting guidelines.

## Results

Twenty-five patients with 37 tumors were treated with a total of 39 CT-guided percutaneous HDR brachyablation procedures between September 2015 to August 2019. Baseline patient characteristics are summarized in Table 1. Twenty-two (88.0%) patients had metastatic lung tumors, 2 (8.0%) had primary NSCLC, and 1 (4.0%) had locally recurrent NSCLC. The most common histologies were renal cell carcinoma (n = 6, 24%), NSCLC (n = 5, 20%), and soft tissue sarcoma (n = 5, 20%). Among patients with pulmonary metastasis, 8 (36.3%) had extra-thoracic disease. With the exception of one, all tumors had not received any prior local treatment. Twenty-three (92.0%) patients received at least one prior systemic therapy or local treatment for tumors in other areas of the lung: systemic therapy (72.0%), radiotherapy (56.0%), or IGTA (32.0%).

**Table 1:** Baseline patient characteristics

Age, mean (SD)	66 (11.6)
Gender, n (%)	
Female	9 (36%)
Male	16 (64%)
ECOG performance status, n (%)	
0	10 (40%)
1	15 (60%)
2 +	0 (0%)
Lesion type, n (%)	
Primary NSCLC	2 (8%)
Locally recurrent	1 (4%)
Metastasis	22 (88%)
Histology, n (%)	
Renal cell carcinoma	6 (24%)
NSCLC	5 (20%)
Soft tissue sarcoma	5 (20%)
Gynecological	2 (8%)
Hepatocellular carcinoma	2 (8%)
Other^a^	5 (20%)
CTV volume (cc), mean (SD)	11.6 (12.4)
Lesion location^b^, n (%)	
Ultra-central	20 (54.1%)
Parenchymal target	13 (65%)
Hilar/nodal target	7 (35%)
Central	9 (24.3%)
Peripheral	8 (21.6%)
Prior therapy for different lung tumors, n (%)	
Systemic therapy	18 (72%)
Lung radiation (EBRT or brachytherapy)	14 (56%)
Minimally invasive procedure	8 (32%)
None	2 (8%)

SD, standard deviation; ECOG, Eastern Cooperative Oncology Group; NSCLC, non-small cell lung cancer; CTV, clinical target volume; EBRT, external beam radiotherapy

^a^Other histology included colorectal cancer, salivary gland tumors, thyroid cancer, carcinoid tumor

^b^Per-lesion basis (n = 37), all other results are reported on per-patient basis (n = 25)

Of 37 treated tumors, 20 (54.1%) were ultra-central and 9 (24.3%) were central, respectively. The average CTV was 11.6cm^3^ (SD 12.4, range 0.57–62.8). Mean CTV V125% and V150% were 81.2% (SD 11.4) and 70.7% (SD 13.1), respectively. The mean lung dose to the ipsilateral lung was 2.17 Gy (SD 1.53). Mean V5Gy and V20Gy to the ipsilateral lung were 7.84% (SD 8.32) and 0.85% (1.06). Maximum dose to the most irradiated 2 cc to the heart, esophagus, trachea/proximal bronchus, and chest wall were 4.58 Gy (SD 4.95), 1.02 Gy (SD 1.86), 2.85 Gy (SD 3.61), and 1.98.Gy (SD 4.13), respectively.

**Fig. 2:**
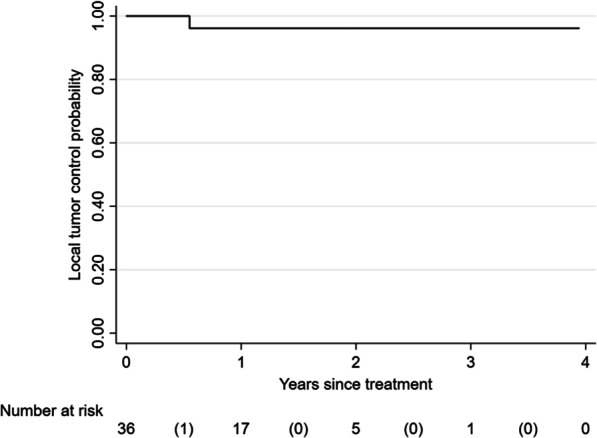
Kaplan–Meier curve for local control

**Fig. 3:**
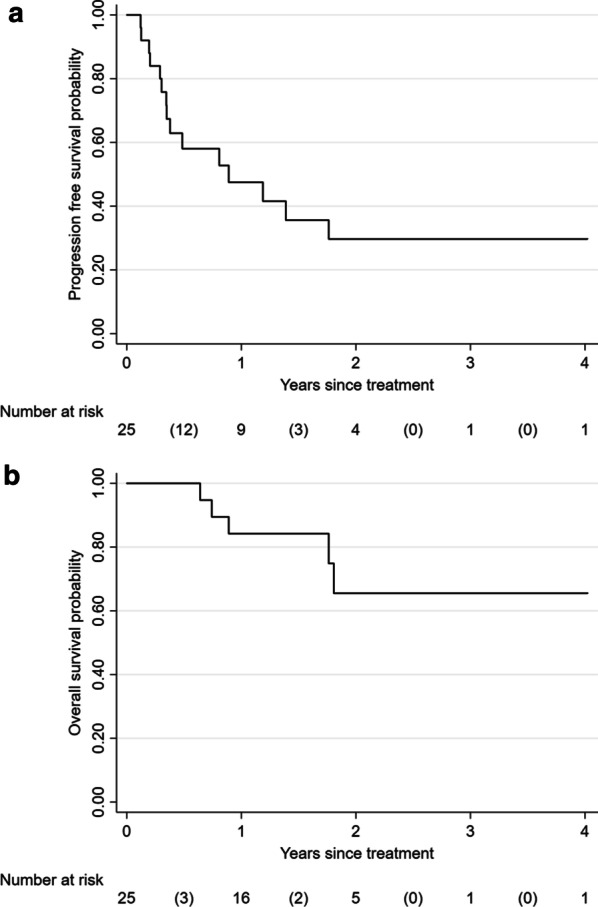
**A** Kaplan–Meier curve for progression free survival. **B** Kaplan–Meier curve for overall survival

Median follow-up time was 19 months (range 3–48). Twenty-one patients representing 33 tumors were evaluable for response assessment. Six (18.2%) of 33 tumors exhibited complete local response, 15 (45.5%) partial response, and 11 (33.3%) stable disease. One tumor (3.0%) demonstrated local progression. The HDR prescription dose to this tumor had been decreased to minimize the risk of injuring to the central airway and mediastinal structures due to prior exposure to external beam radiation. Two- and 3- year LC were 96.2% (Fig. 2). No clinical or treatment covariates were associated with LC. Thirteen (52.0%) patients developed systemic disease progression after treatment, 8 in other areas of the lung and 5 outside. The 2– and 3-year PFS and OS rates were 29.7% and 65.5%, respectively (Fig. 3A, B).

Table 2 summarizes procedural complications and treatment-related toxicities. Thirteen of 39 (33.3%) procedures were associated with minor pneumothoraces requiring no intervention, 1 (2.5%) led to a minor radiographic pulmonary hemorrhage requiring no intervention, and 4 (10.3%) were associated with a major pneumothorax requiring chest tube insertions. All procedural complications resolved within 24 h from treatment. Twenty-two patients representing 32 lesions had data available for acute toxicity evaluations. Eighteen (81.8%) patients did not experience acute toxicities. Two patients developed grade 1 acute toxicities (cough and pain), and 2 patients experienced grade 2 toxicity (pneumonitis and pain). Post-treatment toxicities were limited to (ultra)central tumors, and resolved within 1–2 weeks. Eighteen patients with 30 lesions had available late toxicity data, none of whom developed late treatment-related toxicities. One patient experienced mild dyspnea on exertion 5 months after treatment, which was attributed to prior smoking and multiple surgical lung resections and microwave ablations.

**Table 2:** Rates of procedural complications and treatment-related toxicities following CT-guided HDR interstitial brachytherapy ablation

Procedural complication rate^a^	Total procedures (n = 39)
Minor pneumothorax	13 (33.3%)
Major pneumothorax	4 (10.3%)
Pulmonary hemorrhage^b^	1 (2.5%)
Acute toxic events	Total evaluable patients (n = 22)
Grade 0	18 (81.8%)
Grade 1	2 (9.1%)
Grade 2	2 (9.1%)
Grade ≥ 3	0 (0%)
Late toxic events	Total evaluable patients (n = 18)
Grade 0	18 (100%)
Grade 1	0 (0%)
Grade 2	0 (0%)
Grade ≥ 3	0 (0%)

^a^All procedural complication rates self-resolved within 24 h

^b^Pulmonary hemorrhage was grade 1

## Discussion

This study showed that CT-guided percutaneous HDR brachyablation yields high long-term tumor LC with low rates of procedural and treatment-related toxicities with a median follow-up time of 19 months. With 78.4% of tumors being ultra-centrally or centrally located, the 2- year LC was 96.2%. Only 4 patients developed transient acute toxicities, all of which were grade 1–2, and no patients developed late toxicities. However, about half of patients developed disease progression outside the irradiated area and2-year PFS was 29.7%, underscoring a multi-disciplinary approach whenever managing patients with any degree of metastatic disease.

Brachyablation for lung tumors was first introduced outside the United States with a focus on low-dose rate (LDR) approaches. A meta-analysis of 296 patients from 5 clinical trials with advanced lung cancer patients reported the addition of LDR iodine-125 brachytherapy to chemotherapy was associated with improved tumor response (RR = 1.85, 95% CI 1.54–2.22, *P* < 0.001) and disease control (RR = 1.19, 95% CI 1.10–1.29, *P* < 0.001); these gains were not associated with improvement in OS [15]. Although LDR is associated with greater sparing of normal late-responding tissues such as the lung, it has also been associated with less tumor cell killing [16].

Interest in HDR brachyablation in the management of pulmonary tumors has recently grown; yet, to our knowledge this is the first report from an institution in the United States. The findings in our analyses support related experiences outside the United States that similarly reported high rates of long-term LC with a favorable safety profile in patients with central lung tumors. These include data from a phase I study in Germany reported by Ricke et al. in 2004 that demonstrated 97% LC in 15 patients with 30 lung tumors treated to at least 20 Gy in a single fraction with 5 + months of median follow-up [17]. Peters et al. in 2008 demonstrated 1-year LC of 91% after treating 30 patients with 83 primary and secondary lung malignancies to at least 20 Gy in a single fraction in a single-arm phase 2 study [18].

The experience of a third group in Germany, reported by Tselis et al. in 2011, was reported in a retrospective analysis on 55 patients with 60 tumors who received a median dose of 20 Gy (range 7–32) with multi-fractionated treatments; approximately half of their patients received twice-daily fractions of median 6 Gy per fraction, whereas the other half received once daily fractions with a median of 8 Gy per fraction [19]. After a median follow-up of 14 months, 2-year LC rates were 82% for metastatic tumors, and 79% for primary and locally recurrent intrathoracic lesions. Although high, the LC rates from this retrospective study were relatively lower than those from our study. Differences in control may be due to dose fractionation as clinical evidence suggests that tumor cell killing with single fraction HDR radiation may be more effective for late-responding tissues compared to a multi-fractionated regimen [16, 20]. Furthermore, early studies from SBRT for NSCLC showed delivering BED_3_ > 100 Gy to tumor was significantly correlated with improved LC, which some patients in this study may not have received and led to the relatively lower LC rates compared to our study [21, 22].

By nature of its “inside-out” radiation delivery, brachyablation maximizes the therapeutic ratio by delivering tumoricidal doses with a sharp dose drop-off outside the tumor; therefore, potentially improving upon the dose distribution compared to SBRT [23]. Although early studies from small (< 5 cm in diameter) peripheral early-stage NSCLC and metastatic lung tumors demonstrated 5-year LC of ~ 92–93% [22, 24], SBRT can potentially cause severe toxicities when treating centrally located tumors. In a landmark phase 2 trial, 2-year freedom from severe (grade 3–5) toxicity of only 54% for patients with central tumors compared to 83% for those with peripherally located tumors after receiving 60–66 Gy in 3 fractions [6]. More acceptable toxicity rates were achieved by delivering the total radiation dose over a greater number of treatment fractions [25]. The risk for developing severe or fatal toxicities still poses a challenge when treating ultra-central tumors with SBRT [4, 5, 26, 27]. It is notable that while the majority of patients in our study had (ultra)central lesions, favorable LC rates were achieved with minimal morbidity after just a single fraction of high dose radiation.Moreover, doses to the normal ipsilateral lung and critical mediastinal structures were low.

HDR brachyablation has an advantage over SBRT due to the relatively smaller volume of tissue radiated. Respiratory motion introduces uncertainty to tumor localization during SBRT treatment planning. Despite various strategies to manage respiratory motion, a margin of normal lung tissue surrounding the target is added during SBRT to account for this uncertainty. In contrast, brachytherapy catheters are directly implanted into the tumor and eliminate uncertainties from respiration [28, 29]. Additionally, due to the increasing utilization of immunotherapy there is interest in reducing volume of normal tissues receiving low doses, in order to minimize immunosuppressive effects. Future comparative dosimetric analyses between HDR brachyablation and SBRT are of high interest.

Incidence of procedural complications in our study was higher compared to previous experiences with HDR brachyablation but comparable to reports with IGTA [17–19, 30–33]. In our study, minor pneumothorax was associated with 33.3% of procedures (representing 44% of patients) and major pneumothorax in 10.3% of procedures (12% of patients). Prior HDR brachyablation studies reported approximately 12% of patients developing minor pneumothorax and one patient developing a major pneumothorax [18, 19]. Discrepancies in procedural complication rates may be attributed to operator expertise and/or our brachytherapy catheter implantation techniques. Also, all patients in our cohort underwent routine post-procedure surveillance CT scans whereas potentially in other series only chest x-ray was utilized. Detection of small pneumothoraces is much higher with CT as compared to chest X-ray [34, 35]. Tumor access was achieved using a 17-gauge co-axial needle through which a 4F catheter was placed. The needle provided continuous structural support for the catheter to prevent unwanted migration from its initial placement. In comparison, prior brachytherapy studies placed an 18-gauge introducer needle followed by guidewire exchange for 6F angiography sheath and 16-gauge catheter, or placement of traditional 6F brachytherapy plastic catheter with rigid inner obturator. Utilizing a smaller needle gauge allowed better access to smaller and central tumors, including hilar and mediastinal targets. The resultant range of treated tumor volumes was generally smaller compared to related studies [17–19].

The overall lower peri-procedural complications reported in previous HDR brachyablation studies compared to IGTA may be due to differences in biologic effects. Cytotoxic effects from radiation occur over weeks to months whereas IGTA causes instantaneous cell death and necrosis. The less immediate structural changes with radiation may slow tissue reorganization and mitigate the formation of pneumothoraces [16, 30]. Injection of hydrogel biosealant upon needle removal has also shown to decrease chest tube insertion rates during lung biopsy, although pneumothorax rates have varied between studies [36, 37].

Interstitial HDR brachyablation may also overcome some limitations faced with IGTA.  IGTA is better suited for peripheral, small tumors, and generally avoided for central tumors near mediastinal or large bronchial structures, diaphragm, or larger blood vessels HDR brachyablation can safely and effectively treat central and ultra-central tumors that were not possible or potentially ineffective with IGTA. Not only can brachyablation better protect neighboring organs with its sharp dose gradient, but 3D computer-generated radiation TPS calculates an optimal dose distribution prior to treatment delivery and can minimize the delivery of excessively high doses to critical nearby structures. Precise dose measurements cannot be calculated with IGTA since several factors impacting energy deposition; including thermal conductivity, impedance, and perfusion; cannot be accounted at the time of the procedure.

An intriguing finding in this report is that LC following HDR brachyablation was not shown to depend on tumor size as it does with IGTA [18, 19]. This may be related to the use of a TPS that ensures adequate coverage of each tumor to its surface. This series demonstrated high rates of LC at 2 years for tumors with an average volume of 11.6cm^3^ (range 0.57–62.8), while LC declines when treating tumor volumes greater than 3 cm in diameter with IGTA [9, 31, 38, 39]. from the use of multiple catheters to attain optimal dosimetry to ensure adequate tumor coverage and deliver non-uniform doses that can facilitate the delivery of very high doses to certain regions of the tumor while minimizing exposure to critical structures that may juxtapose on another edge of the target. This provides the option to intentionally deliver treatment in a non-homogenous manner which can boost intratumoral areas that may exhibit more radioresistant properties that may be identified by hypoxic radiographic signatures [20, 40].

This study has several limitations that deserve mention as the findings may not be widely generalizable. This study cohort was highly selected and represented a small and heterogeneous cohort. The outcomes of procedural studies often rely on technical and institutional expertise, thus complication rates may be higher in less experienced centers. The use of CTCAE criteria has been associated with under-reporting acute and late toxicities by physicians [41, 42]. Likewise, assessment of tumor response is subject to bias especially when radiation-related changes or other pathologies are present. In this study, tumor response was independently reviewed by two radiation oncologists and a thoracic radiologist using a prospectively validated criteria to mitigate this risk, and there was high agreement between physicians. As familiarity and experience with brachytherapy grows, further studies with larger cohort of central lung tumors are warranted. Furthermore, managing pulmonary tumors with HDR brachyablation should be made in a multi-disciplinary setting and performed in a high-volume brachytherapy facility. Engagement with Interventional Radiology physicians is highly recommended.

## Conclusion

This study demonstrates that percutaneous HDR brachyablation is a promising therapeutic option to eradicate centrally and ultra-centrally located primary and metastatic lung tumors. Larger studies are needed to confirm its safety and efficacy as well as future comparisons to stereotactic body radiotherapy and other ablative techniques are warranted to expand multi-disciplinary management options.

## Supplementary Information

**Additional file 1**. Derivation of biologic effective dose and equivalent dose in 2Gy per fraction.

## Data Availability

The datasets used and/or analyzed during the current study are available from the corresponding author on reasonable request.

## Abbreviations

AAPM: The American Association for Physicists in Medicine
BED: Biologic effective dose
CT: Computed tomography
CTCAE: Common terminology criteria for adverse events
CTV: Clinical target volume
EQD2: Equivalent dose in 2 Gy per fraction
HDR: High-dose rate
IGTA: Image-guided thermal ablation
LC: Local control
LDR: Low-dose rate
MWA: Microwave ablation
NSCLC: Non-small cell lung cancer
OAR: Organ-at-risk
OS: Overall survival
PFS: Progression free survival
RECIST: Response evaluation criteria for solid tumors
RFA: Radiofrequency ablation
RR: Relative risk
SBRT: Stereotactic body radiotherapy
SD: Standard deviation
TPS: Treatment planning system

## Data access and integrity

The authors declare that they had full access to all the data in this study and the authors take complete responsibility for the integrity of the data and the accuracy of the data analysis.
