# Supplementary material for: Outcomes with multi-disciplinary management of central lung tumors with CT-guided percutaneous high dose rate brachyablation
Source: Radiat Oncol. 2021 Jun 7;16:99. doi: 10.1186/s13014-021-01826-1 (PMC8186067; doi:10.1186/s13014-021-01826-1)
Supplement: Supplementary file 1 — Additional file 1. Derivation of biologic effective dose and equivalent dose in 2Gy per fraction. [file 13014_2021_1826_MOESM1_ESM.docx]

**Appendix.** Total radiation dose comparison using biologic effective dose (BED) and equivalent dose in 2Gy per fraction (EQD2)

Total radiation dose delivered to target during radiotherapy was thought to be an important factor to consider for oncologic outcomes and toxicity development. A common numeric score, the biologic effective dose (BED), was calculated to compare total radiation dose delivered across various dose fractionation schemas. The equation for BED is outlined below:

(Eq. A.1) $BED=nd[1+ \frac{d}{\alpha/\beta}$]

BED is biologic effective dose. *n* is number of radiation treatment fractions. *d* is dose per fraction. α/β is the dose where linear and quadratic cell killings are equal.

Early responding tissues to radiation have higher α/β ratios while late responding tissues have lower values. Lung tissue is considered a late-responding tissue, and an α/β ratio of 3 was used for our calculations (denoted as BED_3_).

Another related and common numeric score to compare total radiation dose delivered by various dose fractionation schemas is the equivalent dose in 2Gy per fraction (EQD2). The equation for EQD2 is outlined below:

(Eq. A.2) $EQD2=\frac{BED}{1+ \frac{2}{\alpha/\beta}}$

EQD2 is equivalent dose in 2Gy per fraction. BED is biologic effective dose. α/β is the dose where linear and quadratic cell killings are equal.
